# Serum Lutein is related to Relational Memory Performance

**DOI:** 10.3390/nu11040768

**Published:** 2019-04-02

**Authors:** Corinne N. Cannavale, Kelsey M. Hassevoort, Caitlyn G. Edwards, Sharon V. Thompson, Nicholas A. Burd, Hannah D. Holscher, John W. Erdman, Neal J. Cohen, Naiman A. Khan

**Affiliations:** 1 Neuroscience Program, University of Illinois at Urbana-Champaign, Champaign, IL 61801, USA; cannava2@illinois.edu (C.N.C.); njc@illinois.edu (N.J.C.); 2 Beckman Institute for Advanced Science and Technology, University of Illinois at Urbana-Champaign, Champaign, IL 61801, USA; kelseyhassevoort@gmail.com; 3 Center for Brain Plasticity, University of Illinois at Urbana-Champaign, Champaign, IL 61801, USA; 4 Division of Nutritional Sciences, University of Illinois at Urbana-Champaign, Champaign, IL 61801, USA; cgedwar2@illinois.edu (C.G.E.); svthomp2@illinois.edu (S.V.T.); naburd@illinois.edu (N.A.B.); hholsche@illinois.edu (H.D.H.); jwerdman@illinois.edu (J.W.E.J.); 5 Department of Kinesiology and Community Health, University of Illinois at Urbana-Champaign, Champaign, IL 61801, USA; 6 Department of Food Science and Human Nutrition, University of Illinois at Urbana-Champaign, Champaign, IL 61801, USA; 7 Department of Psychology, University of Illinois at Urbana-Champaign, Champaign, IL 61801, USA

**Keywords:** obesity, hippocampus, nutrition, overweight, carotenoids

## Abstract

Dietary carotenoids, plant pigments with anti-oxidant properties, accumulate in neural tissue and are often found in lower concentrations among individuals with obesity. Given previous evidence of negative associations between excess adiposity and memory, it is possible that greater carotenoid status may confer neuroprotective effects among persons with overweight or obesity. This study aimed to elucidate relationships between carotenoids assessed in diet, serum, and the macula (macular pigment optical density (MPOD)) and relational memory among adults who are overweight or obese. Adults aged 25–45 years (*N* = 94) completed a spatial reconstruction task. Task performance was evaluated for accuracy of item placement during reconstruction relative to the location of the item during the study phase. Dietary carotenoids were assessed using 7-day diet records. Serum carotenoids were measured using high-performance liquid chromatography. Hierarchical linear regression analyses were used to determine the relationship between carotenoids and task performance. Although initial correlations indicated that dietary lutein, beta-carotene, and serum beta-carotene were positively associated with memory performance, these relationships were not sustained following adjustment for age, sex, and BMI. Serum lutein remained positively associated with accuracy in object binding and inversely related to misplacement error after controlling for covariates. Macular carotenoids were not related to memory performance. Findings from this study indicate that among the carotenoids evaluated, lutein may play an important role in hippocampal function among adults who are overweight or obese.

## 1. Introduction

Overweight and obesity are conditions that increase metabolic risk and are characterized by a body mass index (BMI) greater than 25 kg/m^2^ [1]. Obesity can lead to a variety of metabolic and cardiovascular concerns such as type 2 diabetes, non-alcoholic fatty liver disease, and cardiovascular disease [1]. According to the National Health and Nutrition Examination Survey (NHANES) data from 2015–2016, approximately 40% of adults in the United States have obesity, with women having a slightly higher prevalence than men (41.1% vs. 37.9%) [1]. In addition to the cardiometabolic concerns that accompany obesity, excess fat mass or adiposity, as well as the associated metabolic complications, have been associated with poorer cognitive function and brain structure [2]. One specific brain region thought to be affected by obesity and other associated disorders is the hippocampus [2,3,4]. The hippocampus is a highly plastic region of the brain, which may explain why hippocampal-dependent memory function can be susceptible to behavior modulation through environmental factors [5,6]. Obesity, as well as a “western” diet characterized by high saturated fat and added sugar intake, has been related to poorer hippocampal function [3,4]. However, research examining the influence of specific dietary components on relational memory among individuals with overweight or obesity has been limited.

Relational memory is a hippocampal-dependent process that involves the flexible binding of arbitrary elements within an episode, and the subsequent reactivation of these relations [7]. In a practical sense, relational memory allows us to put a name to a face or re-tell the story of a trip in any order one chooses. One approach for assessing relational memory is to use a spatial reconstruction task. Spatial reconstruction tasks require participants to return objects to locations they have previously studied. This task design requires the participant to encode the arbitrary relationships between objects and locations (or other objects) within each trial and subsequently use these bindings to reconstruct the studied display successfully. Previous studies have shown that obesity and diet are associated with relational memory performance; however, this has not yet been thoroughly investigated [4,8].

Carotenoids have been recently found to have relevance for hippocampal-dependent memory performance [7]. Carotenoids are plant pigments that represent red, yellow, and orange color, and can be found in egg yolks and a variety of fruits and vegetables such as avocados, carrots, sweet potatoes, spinach, and other leafy green vegetables [9]. Previous studies have shown that, among the numerous carotenoids in nature, only a handful accumulate in human neural tissue. These include lutein, beta-carotene, beta-cryptoxanthin, and zeaxanthin, all of which accumulate in all cortices and the hippocampus in humans and non-human primates [10,11,12]. However, lutein accumulation in neural tissue is up to 5-fold greater than other carotenoids, inferring a potentially unique role for this carotenoid in cognitive function and brain health [10]. Further, lutein, its stereoisomer, zeaxanthin, and the lutein intermediate *meso*-zeaxanthin, collectively belonging to a group known as xanthophylls, selectively accumulate in the macula of the human eye. The structural properties of xanthophylls allow them to serve as blue light filters and antioxidants in the eye and protect retinal tissue from photo-oxidative damage [9,13,14]. Opportunely, the macular concentration of lutein, zeaxanthin, and *meso*-zeaxanthin can be non-invasively assessed as macular pigment optical density (MPOD) [15]. Macular xanthophylls have previously been related to brain carotenoid concentrations in non-human primates [16]. These macular carotenoids, assessed through MPOD, are also positively associated with relational memory performance in children and with intellectual ability and executive function in adults [7,17,18]. Additionally, higher MPOD scores have been associated with better memory performance assessed using a delayed recall task [11,19]. However, given that MPOD represents a composite metric of all three xanthophylls in the retina, it is incorrect to isolate carotenoid-related cognitive benefits to lutein alone. Individual carotenoid quantification in serum provides an opportunity to study the influence of lutein in a manner that would be separable from other carotenoids in vivo. While serum does not provide a direct assessment of neuronal lutein, it provides us with a more precise measure of individual carotenoids than what is determined using MPOD or dietary assessment. In previous studies quantifying serum lutein rather than the composite MPOD measure, it was shown that serum lutein mediated the relationship between the parahippocampal cortex and crystallized intelligence [20]. Serum lutein is also associated with multiple domains of cognition, including memory [11]. However, a potential limitation of relying on serum alone is that the content in serum is transient and may not reflect xanthophyll status in neural tissue. Therefore, additional studies that assess both MPOD and serum carotenoids are necessary to clarify the impact of lutein and other carotenoids on relational memory. To our knowledge, there have been no previous studies investigating associations between both MPOD and serum xanthophylls on relational memory abilities among adults with overweight or obesity. Accordingly, this study aimed to understand whether dietary, serum, or macular levels of carotenoids were associated with relational memory function in adults that are overweight and obese. We hypothesized that macular carotenoids, assessed as MPOD, would be positively associated with relational memory performance. Additionally, we anticipated that a greater serum concentration of lutein would be associated with greater relational memory performance.

## 2. Materials and Methods

### 2.1. Participants

Eligible participants were adults aged 25–45 years who were overweight or obese (BMI ≥ 25 kg/m^2^) and no prior history of physician-diagnosed metabolic or gastrointestinal disease (e.g., Crohn’s Disease, Diabetes, CVD, etc.), or neurological or cognitive disorders. Participants were also excluded if they were using any tobacco products.

### 2.2. Ethical Approval

At the first appointment, participants were informed of the overall procedure and written informed consent was obtained before collection of any data. The University of Illinois Institutional Review Board approved consent before recruitment, and the study was conducted following the Declaration of Helsinki.

### 2.3. Procedure

Data were collected over the course of two appointments. At the first appointment, participants were screened using medical history and demographic questionnaires. The second appointment followed a 10-h overnight fast. The participants subsequently underwent adiposity assessment using dual-energy *X*-ray absorptiometry (DXA) (Hologic, Bedford, MA, USA) and IQ was assessed using the Kaufman brief intelligence test-2 (KBIT-2). A spatial reconstruction task was administered to assess hippocampal-dependent relational memory ability. Fasted blood was collected at the conclusion of the appointment and serum lutein levels were determined using high-performance liquid chromatography. All participants were provided with a 7-day diet record to document their regular carotenoid intake, which was completed within one week of relational memory assessment.

### 2.4. Relational Memory Assessment

**Figure 1:**
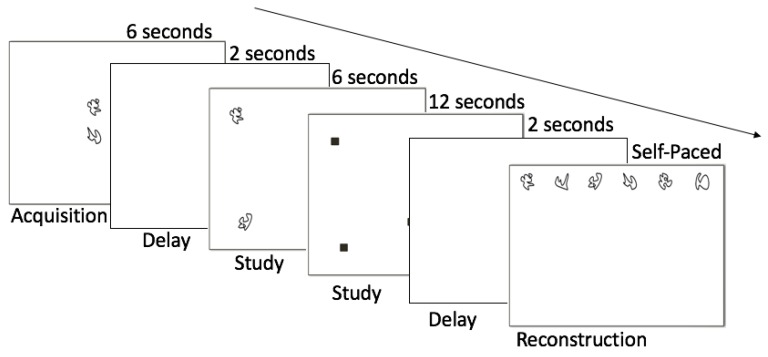
Spatial reconstruction task description.

**Figure 2:**
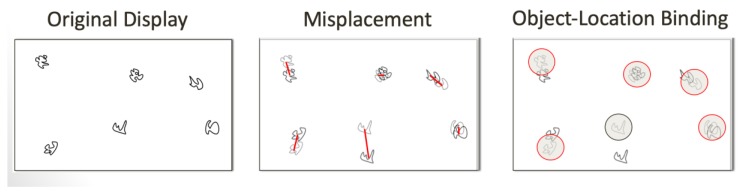
Spatial reconstruction task error metrics.

Hippocampal-dependent relational memory ability was evaluated through a computerized spatial reconstruction task (Figure 1). This task was completed using Presentation Software (Neurobehavioral Systems, Berkeley, CA, USA). Participants were first shown 6 abstract shapes in the center of the screen to introduce the shapes used for the reconstruction phase. After 6 s, the stimuli disappeared and reappeared in a randomized array on the screen. The identities of the objects were then masked by small squares and participants had 18 s to click the boxes and one-by-one learn the locations of each shape. The boxes then disappeared and, after a 2 s fixation, they reappeared at the top of the screen. Participants were instructed to reconstruct the array they previously studied. Reconstruction was self-paced and, once the participant was satisfied with their reconstruction, they moved on to the subsequent trial, for a total of 20 trials (4 blocks of 5). Each participant’s performance was assessed using two error metrics: misplacement and object-location binding (Figure 2). Misplacement was calculated as the average measure of distance (in pixels) between the objects’ studied and reconstructed locations, with a higher score indicating poorer performance. Object-location binding was defined as the number of times the participant correctly placed an item within a pre-defined radius around its studied location. For each trial, a participant received a score of 0–6 for this metric, with a higher score indicating better performance, and performance was averaged across trials. More detailed explanations of these error metrics can be found in Horecka et al. (2018) [21].

### 2.5. Intelligence Assessment

The KBIT-2 was administered by a trained staff member to estimate IQ. The assessment is comprised of 3 subtests: verbal knowledge, matrices, and riddles. Correct answers are given a score of 1 and each subtest score is then transformed to a standardized score normed for ages 4–90 years [22,23]. The verbal knowledge subtest includes 60 questions where the participant chooses which of six images is most associated with a word or question spoken by the researcher. The matrices subtest has 46 logic problems where the participant must choose an image that is most associated with a single stimulus picture or which picture best completes the pattern of a 2 × 2, 2 × 3 or 3 × 3 matrix. The riddle subtest consists of 48 riddles spoken by the researcher where the participant gives a single word response.

### 2.6. Dietary Assessment

All participants recorded regular dietary intake for 7 days in a provided food diary after instruction by a trained staff member. Diet record data were recorded and analyzed using the Nutrition Data System for Research (NDSR) 2015 (Minneapolis, MN, USA) by trained research staff. There was not enough dietary information in the available nutrient databases to extract levels of individual xanthophylls; therefore, dietary lutein and zeaxanthin were ascertained as an aggregate measure (i.e., lutein + zeaxanthin). Consumption amounts of lutein + zeaxanthin, beta-carotene, and beta-cryptoxanthin were extracted from NDSR.

### 2.7. Serum Carotenoid Assessment

Serum carotenoid levels were assessed using high-performance liquid chromatography (HPLC). Serum carotenoids were extracted using 3 consecutive hexane extraction processes using a previously published protocol [24]. Briefly, the hexane layers were combined, dried under nitrogen, taken up into 90% MTBE, 8% methanol, and 2% ammonium acetate in water solution (1.5% solution) and then analyzed for carotenoid concentrations using the Alliance HPLC system (e2695 Separation Module) equipped with 2998 photodiode array detector (Waters, Milford, MA, USA) and a reverse-phase C30 column (4.6 × 150 nm, 3 micron, YMC, Wilmington, NC, USA). Serum levels of carotenoids previously found in human neural tissue were assessed including lutein, zeaxanthin, beta-carotene, and cryptoxanthin.

Carotenoid standards were obtained from Carotenature, Ostermundigen, Switzerland. For quantification, standard curves were run for each carotenoid, and serum carotenoids were quantified by use of the following extinction coefficients (all 1% solution): lutein 2550 in ethanol; zeaxanthin 2540 in ethanol; beta carotene 2592 in hexane; cryptoxanthin 2565 in hexane. The Erdman laboratory routinely participates in the National Institutes for Standards and Testing micronutrient proficiency testing program, and our serum carotenoid values for blinded serum samples consistently were within 1–2 SD of the medians.

### 2.8. Retinal Carotenoid Assessment

MPOD was assessed using a macular densitometer (Macular Metrics Corporation, Rehoboth, MA, USA) via a customized hetero-flicker photometry (cHFP) technique). This two-step process first required participants to focus on a flickering stimulus in their central line of vision, where the macular pigment is at its highest concentration, and 7 degrees parafoveally, where the macular pigment is at its lowest concentration. The stimulus flickered between 460 nm and 570 nm wavelengths at a rate that has been optimized for the width of the null zone for the participant. Participants then adjusted the radiance to identify the point at which they could not detect a flicker (the null flicker zone). MPOD was calculated by subtracting the foveal from the parafoveal log sensitivity measurements after normalizing at 570 nm. More detailed information on the principles behind this technique were described in Wooten, et al. (1999) [25].

### 2.9. Weight Status and Adiposity

Height and weight were measured three times using a stadiometer (model 240; SECA, Hamburg, Germany) and a digital scale (WB-300 Plus; Tanita, Tokyo, Japan) while participants were wearing light clothing and no shoes. Mean height and weight values were used to calculate BMI. Whole body adiposity (%Fat) was measured using a Hologic Horizon W bone densitometer (software version 13.4.2, Bedford, MA, USA) DXA scanner.

### 2.10. Statistical Analysis

All statistical analyses were conducted using SPSS 2016 v24 (IBM Corp., Armonk, NY, USA). Normality was assessed using the Shapiro-Wilk test and variables that did not display normal distribution were log transformed. Pearson’s correlations were run to determine initial relationships between hippocampal task performance, carotenoids, MPOD, Age, Sex, and %Fat. Hierarchical linear regression modeling was used to further investigate the relationships between carotenoids with statistically significant correlations to relational memory metrics. Step 1 of each regression model included covariates that were found to be significantly related to the dependent, relational memory, variable via bivariate correlations. Separate step 2′s were conducted for each carotenoid found to be related to the respective dependent variable from bivariate correlations. A one-tailed approach was used due to the positive directionality of our hypothesis and outcomes seen in the previous literature [17,18,20,26].

## 3. Results

Descriptive statistics for participant demographics can be found in Table 1.

Bivariate correlations revealed that misplacement was positively related to age (*r* = 0.33, *p* = 0.001) and %Fat (*r* = 0.19, *p* = 0.03), and inversely related to IQ (*r* = −0.37, *p* < 0.001). Further, misplacement was negatively associated with dietary lutein + zeaxanthin (*r* = −0.22, *p* = 0.02) and serum lutein (*r* = −0.25, *p* = 0.005) concentrations. Similarly, dietary and serum beta-carotene were negatively associated with misplacement (*r* = −0.24, *p* = 0.01; *r* = −0.20, *p* = 0.03). Object-location binding was negatively related to age (*r* = −0.25, *p* = 0.007) and positively related to IQ (*r* = 0.32, *p* = 0.001). Object-location binding was not associated with %Fat (*r* = −0.11, *p* = 0.1), likely due to the decreased range of performance. Additionally, object-location binding was positively associated with dietary lutein + zeaxanthin (*r* = 0.21, *p* = 0.02), serum lutein (*r* = 0.22, *p* = 0.02), and dietary beta-carotene (*r* = 0.20, *p* = 0.03). No other carotenoids were statistically significantly related to relational memory measures in either diet or serum (all *p*’s > 0.09).

MPOD was not related to relational memory measures (all *p*’s > 0.4), nor was it related to dietary lutein + zeaxanthin (*p* > 0.1). MPOD was, however, related to serum lutein (*r* = 0.30, *p* = 0.002), dietary (*r* = 0.20, *p* = 0.03) and serum (*r* = 0.25, *p* = 0.007) beta-carotene, serum cryptoxanthin (*r* = 0.29, *p* = 0.007), and serum zeaxanthin (*r* = 0.22, *p* = 0.017). Bivariate correlations are summarized in Table 2.

Misplacement was modeled using hierarchical linear regression modeling with dietary lutein + zeaxanthin, serum lutein, and dietary and serum beta-carotene. Each carotenoid was modeled in a separate step 2. Step 1 of each misplacement model controlled for covariates that were statistically significantly related to misplacement in bivariate correlations (Age, %Fat, IQ).

Statistically significant regressions for misplacement can be found in Table 3. Serum lutein was the only carotenoid significantly related to misplacement after covariate adjustment (β = −0.15, *p* = 0.05). Dietary lutein + zeaxanthin (β = −0.07, *p =* 0.2), dietary beta-carotene (β = −0.07, *p =* 0.2), and serum beta-carotene (β = −0.05, *p* = 0.3) were no longer related after controlling for covariates in step 1.

Object-location binding was modeled with serum lutein, dietary lutein + zeaxanthin, and dietary beta-carotene. Similarly, serum lutein was the only carotenoid that was still statistically significantly related to object-location binding after controlling for covariates in step 1 (β = 0.16, *p* = 0.05). Dietary lutein + zeaxanthin (β = 0.10, *p =* 0.1), and dietary beta-carotene (β = 0.08, *p =* 0.2) were no longer related to object-location binding after covariates were accounted for. This model is described in Table 4.

## 4. Discussion

The present work examined the relationship between carotenoids in the macula, diet, and serum, and their relationship with hippocampal-dependent relational memory performance. Given previous literature indicating that lutein disproportionately accumulates in neural tissue, including the hippocampus, we anticipated that serum and macular lutein concentrations, in particular, would be related to relational memory. Herein, the results indicated that higher serum, but not macular, lutein concentrations were positively associated with greater relational memory performance on a spatial reconstruction task. Although dietary lutein + zeaxanthin and both dietary and serum beta-carotene were correlated with performance, these relationships did not persist after covariate adjustment. Taken together, these findings provide additional evidence that serum carotenoid status may impact memory performance among adults who are overweight or obese. 

There are many potential roles lutein may play in the brain. One proposed mechanism, which is relevant in our sample, is that lutein can modulate inflammatory and oxidative stress pathways [13,14]. Participants with overweight or obesity are more susceptible to oxidative and inflammatory stress due to the higher levels of chronic inflammation associated with excess adipose tissue [27,28]. Inflammation and oxidative stress can be mitigated by fruit and vegetable intake, foods that are often rich in carotenoids [27]. Inflammation is detrimental to hippocampal function, specifically by inhibiting long term potentiation, the molecular mechanism for memory formation [29]. In the retina, lutein is protective against age-related macular degeneration by reducing oxidative stress [13,14]. Thus, we hypothesize that lutein may play a similar role in the hippocampus.

Previous studies have shown that elevated serum lutein concentrations are positively associated with multiple realms of cognition, particularly in brain regions where lutein is known to deposit. A recent MRI study found that the parahippocampal cortex mediates the relationships between serum lutein concentrations and fluid intelligence [20]. Additionally, serum lutein concentrations were positively related to delayed recall memory task performance, controlled oral word association tests, and the Weshler adults intelligence scale-III similarities subtest [11]. Vishwanathan et al. did not observe similar relationships with delayed recall and serum lutein + zeaxanthin in their sample [19]. Our results in comparison to these studies indicate that lutein, in particular, may impact cognition in participants with overweight or obesity. When lutein is assessed as a combined metric (dietary, MPOD, serum L + Z), significant associations have not been found. Our results, however, display a strong association with lutein assessed independently of zeaxanthin.

Contrary to our *a priori* hypothesis, macular xanthophylls, assessed by MPOD, were not related to relational memory performance. This was surprising given previous work linking MPOD to relational memory in children, and in older adults which has shown positive associations between other memory forms (e.g., delayed recall) and MPOD [19]. However, our finding may differ from the previous literature due to the weight status of our participants. It is thought that increased adipose tissue, owing to its capacity to store carotenoids, may limit carotenoid availability for other tissues (e.g., retina), which may contribute to this disparity. Though the current body of literature has displayed positive relationships between MPOD and relational memory or executive function, these samples were mainly healthy weight [7,18,26,30,31]. Nevertheless, our team has previously shown that MPOD is associated with intellectual abilities among persons who are overweight or obese [17]. Therefore, it is possible that the relationship between MPOD and cognitive function among persons with overweight or obesity may be domain-dependent. While previous adult studies have displayed relationships with memory function and MPOD, the memory assessments used primarily assessed item-memory and therefore may not have depended on the hippocampus to the extent the spatial reconstruction task does [18,26,30,31]. Additionally, we may have failed to observe the relationship between MPOD and memory due to limitations in the technique we used to assess MPOD. Previous work has shown that, while macular pigmentation is densest at the foveal pit at two sites, we were unable to assess the complete spatial distribution of xanthophylls in the macula, thus, limiting our ability to link macular lutein to memory performance. Additional research studies examining the impact of the spatial profiles of the xanthophylls in the macula on memory function are necessary to characterize the impact of macular lutein to hippocampal function comprehensively. Nevertheless, this work suggests that serum lutein is unique among carotenoids in its relationship with memory. Future studies should assess both blood and macular carotenoids to further elucidate this relationship in a larger and more diverse population. 

While we found significant correlations between memory performance and serum lutein concentrations, there are some limitations worth considering. While spatial reconstruction tasks have been shown to elicit the hippocampus, hippocampal-dependent cognition can also be assessed via other tasks/paradigms that could inform the specific memory processes that benefit from lutein. Second, relative to a previously studied Midwest sample, our serum lutein concentrations were lower when compared to the population average (0.28 µmol/L ± 0.13 versus 0.129 µmol/L ± 0.06) [32]. This, however, may be explained by the higher BMI of our sample. Finally, this study design was cross-sectional, providing no insights into the causal mechanisms that may underlie the carotenoid and relational memory relationship. Intervention studies are necessary to understand whether improvement in lutein status does, in fact, positively influence hippocampal-dependent relational memory performance.

## 5. Conclusions

This study aimed to understand the relationship between dietary, serum, and macular carotenoids, and relational memory. Our results revealed that serum lutein was significantly related to two metrics of relational memory performance even after adjusting for significant covariates. While this study was correlational, it lays the groundwork for subsequent research in this area. Further intervention studies where carotenoids are assessed in the macula and serum must be conducted to better understand this relationship, particularly in participants withoverweight or obesity.

## Figures and Tables

**Table 1:** Participant characteristics and memory performance.

Variable	Mean ± SD
Sex, F/M	45, 54
Age, years	34.9 ± 6.1
BMI, kg/m^2^	33.3 ± 6.6
Fat, %	40.2 ± 8.42
Intelligence Quotient	107 ± 12.1
Macular Pigment Optical Density	0.438 ± 0.20
Dietary Lutein + Zeaxanthin, mcg/day	2283 ± 3382
Serum Lutein, µmol/L	0.129 ± 0.06
Misplacement, pixels	219.3 ± 77.0
Object-location binding	2.67 ± 0.83

**Table 2:** Bivariate correlations (Pearson’s *r*) between relational memory, participant characteristics, and carotenoids.

	Misplacement	Object-Location Binding
Age	0.33 **	−0.25 **
Sex	−0.17	0.10
%Fat	0.19 *	−0.11
IQ	−0.37 **	0.32 **
Dietary L + Z	−0.21 *	0.21 *
Dietary Beta-Carotene	−0.24 **	0.20 *
Dietary Beta-Cryptoxanthin	−0.06	0.05
MPOD	−0.110	0.083
Serum Lutein	−0.266 **	0.223 *
Serum Zeaxanthin	−0.027	0.002
Serum Beta-Carotene	−0.202 *	0.137
Serum Cryptoxanthin	−0.103	0.076

* *p* < 0.05, ** *p* < 0.01.

**Table 3:** Hierarchical linear regression modeling of misplacement and carotenoids.

Step & Variable		β	ΔR^2^
Step 1	Age	0.179 **	0.142 **
	%Fat	0.019	
	IQ	−0.363 **	
Step 2	Serum Lutein	−0.152 *	0.207 *

* *p* < 0.05, ** *p* < 0.01.

**Table 4:** Hierarchical linear regression modeling of object-location binding and carotenoids.

Step & Variable		β	ΔR^2^
Step 1	Age	0.290 **	0.142 **
	IQ	−0.197 *	
Step 2	Serum Lutein	0.159 *	0.166 *

* *p* < 0.05, ** *p* < 0.01.
